# Timing of breast cancer surgery in relation to menstrual cycle phase: no effect on 3-year prognosis: The ITS Study

**DOI:** 10.1038/sj.bjc.6604120

**Published:** 2007-12-18

**Authors:** H Thorpe, S R Brown, J R Sainsbury, T J Perren, V Hiley, M Dowsett, A Nejim, J M Brown

**Affiliations:** 1 Clinical Trials Research Unit, Leeds Institute of Molecular Medicine, University of Leeds, 17 Springfield Mount, Leeds LS2 9NG, UK; 2 Department of Surgery, University College London, The Medical School Building, 74 Huntley Street, London WCIE 6AU, UK; 3 CRUK Cancer Medicine Research Unit, St James's University Hospital, Beckett Street, Leeds LS9 7TF, UK; 4 Academic Department of Biochemistry, Royal Marsden Hospital, Fulham Road, London SW3 6JJ, UK; 5 Airedale General Hospital, Skipton Road, Steeton, Keighley BD20 6TD, UK

**Keywords:** breast cancer, timing of surgery, menstrual cycle phase, survival, prospective study

## Abstract

The effect of breast cancer surgery timing during the menstrual cycle on prognosis remains controversial. We conducted a multicentre prospective study to establish whether timing of interventions influences prognosis. We report 3-year overall and disease-free survival (OS/DFS) results for ‘primary analysis’ patients (regular cycles, no oral contraceptives within previous 6 months). Data were collected regarding timing of interventions in relation to patients’ last menstrual period (LMP) and first menstrual period after surgery (FMP). Hormone profiles were also measured. Cox's proportional hazards model incorporated LMP in continuous form. Exploratory analyses used menstrual cycle categorisations of Senie, Badwe and Hrushesky. Hormone profiles with LMP and FMP data were also used to define menstrual cycle phase. Four hundred and twelve ‘primary analysis’ patients were recruited. Three-year OS from first surgery was 90.7, 95% confidence interval (CI) [87.9, 93.6%]. Menstrual cycle according to LMP was not statistically significant (OS: hazard ratio (HR)=1.02, 95%CI [0.995,1.042], *P*=0.14; DFS: HR=1.00, 95%CI [0.980,1.022], *P*=0.92). Timing of surgery in relation to menstrual cycle phase had no significant impact on 3-year survival. This may be due to 97% of patients receiving some form of adjuvant therapy. Survival curves to 10 years indicate results may remain true for longer-term survival.

Interest in the influence of the menstrual cycle phase at the time of surgery, especially in patients with primary breast cancer, has been the subject of much controversy over the last 20 years. In 1988 Ratajczak *et al* (1988), using a mouse model, showed a relationship between the incidence of postoperative pulmonary metastasis and timing of mammary tumour removal within the reproductive cycle. The following year Hrushesky and co-workers performed a retrospective review of 44 premenopausal women and showed that survival varied according to timing of surgery in the menstrual cycle. They concluded that patients operated on during the perimenstrual period of the cycle had worse disease-free (DFS) and overall survival (OS) than patients who were operated on during other phases of the cycle. Further studies found differing results; some (Badwe *et al*, 1991; Veronesi *et al*, 1994) supported the hypothesis (albeit with different days of heightened risk), others found no effect (Goldhirsch *et al*, 1991; Nathan *et al*, 1993) and one (Sainsbury *et al*, 1991) found an opposite effect, with patients operated on in the follicular phase surviving longer than those operated on in the luteal phase.

All the above studies were retrospective and relied on the patients’ recall of their last menstrual period (LMP) before surgery. This was not available for all patients and its accuracy was not known. It also assumed that ovulation took place 15 days after the first day of menstruation. The subject was reviewed by McGuire (1991) who was critical of the methodology. He demonstrated that changing the definition of duration of the phase of the natural cycle by a couple of days shifted significant numbers of patients from a luteal to a follicular (or vice versa) phase.

In 1992 we instituted the Yorkshire Breast Cancer Group Intervention, Timing and Survival (ITS) study, a multicentre, prospective, observational study of premenopausal women presenting with *de novo* breast cancer, aiming to investigate whether the prognosis for breast cancer patients varies according to the phase of the menstrual cycle during which surgery is performed. Here we report the results of 3-year overall and DFS for the ‘primary analysis’ group. This consisted of those patients who had regular menstrual cycles (i.e. whose duration of menstrual cycle varied by less than 5 days each month) and had not taken the oral contraceptive pill within the previous 6 months before study recruitment in order that hormonal profiles of patients remained as homogeneous as possible.

## MATERIALS AND METHODS

Premenopausal women with suspected operable primary breast cancer were eligible for study entry. Exclusion criteria were previous cancer (except non-melanoma skin cancer or *in situ* cancer of the cervix), pregnancy at the time of diagnosis, having taken hormone replacement therapy within 6 months before diagnosis, screen-detected cases or patients who had had a hysterectomy. Patients were not randomised to undergo interventions at a specified phase of their menstrual cycle and current treatment practice was maintained within each participating centre. Since ITS was a non-randomised observational study, patients identified as ineligible, for example, due to non-malignant tumours, were not followed up. The ITS study received approval from multicentre and local research ethics committees.

Data were collected regarding the timing of interventions (first tumour handling (FTH) and subsequent surgeries) in relation to patients’ menstrual cycle. The date of the LMP before each intervention, hormonal profiles (progesterone, oestradiol, follicle-stimulating hormone and luteinising hormone) at each intervention and the date of the first menstrual period after first surgery (LMP) were collected. Data were also collected regarding adjuvant treatment (chemotherapy, endocrine therapy and/or radiotherapy) administration and site of postoperative radiotherapy, and pathology.

The primary 3-year endpoint was OS, with DFS, local recurrence-free survival and systemic recurrence-free survival at 3 years as secondary endpoints. The key 3-year primary endpoint was OS from first surgery. Longer-term survival will be reported at 10 years.

Due to the uncertainty of the influence of the timing of interventions in relation to the menstrual cycle phase on prognosis, and of the most appropriate categorisation of the menstrual cycle into phases, the primary aim of the ITS study was to identify whether the timing of interventions does indeed affect outcome, and to investigate an optimal interval for surgery. Using only the date of the LMP, patients were therefore categorised into one of seven menstrual cycle groups (0–4, 5–9, 10–14, 15–19, 20–24, 25–29 and 30+ days between LMP and intervention) for the initial analysis. Last menstrual period was also categorised according to the menstrual cycle phase definitions of Senie *et al* (1991) (luteal: days 15–36; follicular: days 0–14), Badwe *et al* (1991) (luteal: days 0–2 and 13–32; follicular: days 3–12) and Hrushesky *et al* (1989) (mid-cycle: days 7–20; perimenstrual: days 0–6 and 21–32). Patients were also categorised as being in either the luteal or follicular phase of their menstrual cycle at the time of intervention using LMP, FMP and hormonal profiles at the time of intervention, along with data regarding menstrual cycle length and variation, in order that patients’ phase of menstrual cycle at the time of intervention could be identified as accurately as possible. This categorisation was performed by an independent expert who was blinded to patients’ outcome.

Since the primary aim of this study was to consider whether the timing of surgery affects prognosis, that is, not a specific comparison of groups, the sample size calculation was based on the number of patients required to investigate the optimal interval of the menstrual cycle. To ensure that each of the above seven menstrual cycle groups had an adequate number of patients (60–65 patients per group) and an adequate number of events for the analysis of the optimal interval (Harrell *et al*, 1985), between 400 and 450 patients were therefore needed to be recruited to the ‘primary analysis’ group. In the event that two phases of the menstrual cycle would be identified as important with regard to the timing of surgery, recruitment of between 300 and 450 patients would be sufficient to detect a clinically relevant difference of at least 10%, with 85% power, for various underlying survival patterns.

Overall survival was defined as time from intervention to death from all causes, and DFS was defined as time from intervention to recurrence or death from all causes. Follow-up beyond 3 years of intervention was censored. Kaplan–Meier curves were calculated to compare OS and DFS by menstrual cycle phase defined using the various categorisations (as described above), and differences in survival between the seven LMP groups of menstrual cycle were tested using the log-rank and Wilcoxon tests. Cox's proportional hazards (PH) model was used to assess the independent influence of timing of intervention in relation to the phase of menstrual cycle on survival, adjusting for Nottingham Prognostic Index (NPI), type of intervention and adjuvant therapies (chemotherapy, radiotherapy and hormone therapy). Analysis incorporated LMP into the Cox model in its continuous form (Altman and Lyman, 1998), and subsequent exploratory analyses considered the categorisations of Senie and co-workers, Badwe and co-workers and Hrushesky and co-workers, and menstrual cycle phases defined using hormone profiles with LMP and FMP data. In order to identify a possible optimal interval of timing of surgery in relation to the phase of menstrual cycle according to LMP, the statistical methods employed by Keefe and Mackie (1991) were used on the key 3-year primary endpoint. The deviance for the Cox PH model where phase of menstrual cycle was considered as a factor variable, using the seven categorisations of LMP, was calculated. The mean value within each of these seven groups was then calculated and substituted for the ordinal factor level in the Cox PH model (i.e. entered into the model as a regressor variable). The difference in deviances of the two models was compared with a *χ*^2^-distribution with five degrees of freedom. By comparing these models we were able to address whether the relationship between menstrual cycle phase at surgery and survival was linear, or whether patients could in fact be categorised into groups with differing probabilities of survival. If an optimal interval were identified for OS, this optimal categorisation would also be investigated in the analysis of DFS.

A two-sided 5% significance level was used for the key primary endpoint of OS from first surgery, and a two-sided 1% significance level was used for all other endpoints (ICH E9 Expert Working Group, 1999). No statistical testing was carried out on any exploratory analyses. All statistical analyses were performed using SAS Version 8.2 (SAS Institute Inc., Cary, NC, USA).

## RESULTS

**Figure 1:**
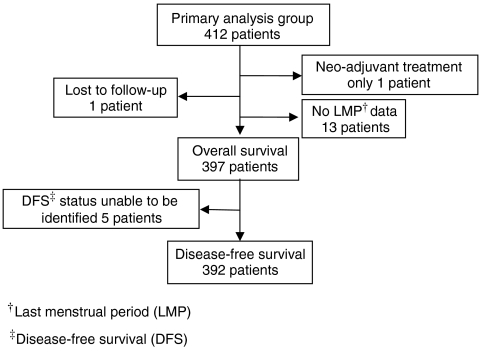
Flow chart of primary analysis group patients for 3-year survival analysis from first surgery.

Six hundred and eleven patients were recruited to the ITS study between February 1993 and December 2000, across 24 UK centres and one Italian centre, and 412 (67.4%) of these patients were in the ‘primary analysis’ group. Table 1 displays baseline characteristics for these primary analysis patients. Median follow-up was 59 months (inter-quartile range (IQR)=[43, 78]) and median age at first surgery was 43 years (IQR=[40, 46]). Seventy-four patients (18.0%) had an excision biopsy as their first surgical intervention, 197 patients (47.8%) had a lumpectomy and 138 patients (33.5%) had a mastectomy. The median time from FTH to first surgical intervention was 17 days (IQR=[10, 26]), and 134 patients (32.5%) required further surgery. No association was observed between timing of first surgical intervention in relation to patients’ menstrual cycle phase and the need for further surgery (data not displayed). Figure 1 shows a breakdown of the primary analysis group patients for 3-year survival from first surgery.

Overall survival from first surgery at 3 years for all primary analysis group patients was 90.7% (95% confidence interval (CI) [87.9, 93.6%]). Table 2 shows the number of deaths and censored values according to the seven LMP groups, under univariate analysis. No statistically significant differences in 3-year OS were identified between the seven LMP groups (log-rank=1.52, df=6, *P*=0.96). Under multivariate analysis, 366 patients were included. Menstrual cycle according to LMP at the time of first surgery and incorporated into the Cox PH model in its continuous form was not found to be a statistically significant independent predictor of 3-year OS (likelihood ratio=2.13, df=1, *P*=0.14) (Table 3). Nottingham Prognostic Index was found to be the only statistically significant factor contributing towards OS (likelihood ratio=29.77, df=1, *P*<0.0001, hazard ratio (HR)=2.34, 95% CI [1.73, 3.18]) under multivariate analysis. Results for OS from FTH were similar.

**Figure 2:**
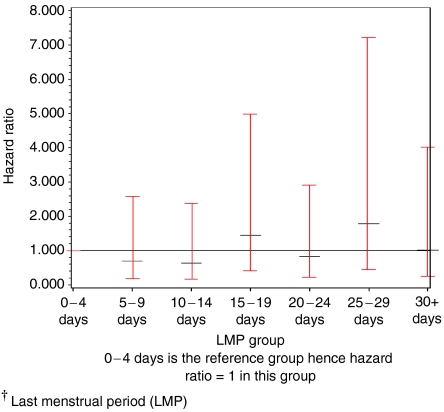
Identifying an optimal interval of the menstrual cycle according to LMP^†^ analysis – HRs and 95% CIs for each LMP group.

In identifying an optimal interval for LMP, Figure 2 displays HRs and 95% CIs for each of the seven LMP groups, with the ‘0–4 days’ group being the reference group. The HRs were shown not to be linear, with the hazards in the ‘5–9 days’, ‘10–14 days’ and ‘20–24 days’ groups being lower in comparison with the ‘0–4 days’ group, while the hazards in the ‘15–19 days’ and ‘25–29 days’ groups were higher. Confidence intervals were however wide and all contained the value one. According to the method of Keefe and Mackie (1991), it was found that there was no significant benefit of fitting LMP as a factored variable compared with a regressor variable (likelihood ratio=2.77, df=5, *P*=0.74), indicating that there may not be a step function relationship between survival and LMP according to the seven-group categorisation. Hence no optimal interval for LMP was identified.

**Figure 3:**
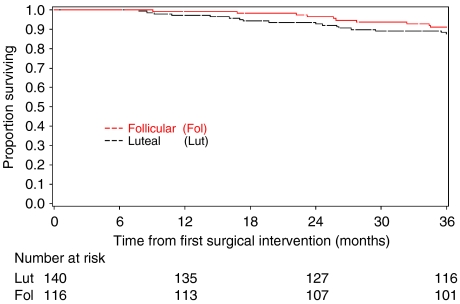
Three-year OS from first surgery according to menstrual cycle phase defined using hormone profiles with LMP and FMP data.

Where menstrual cycle was categorised into the follicular or luteal phase according to hormonal profiles with LMP and FMP data, of the 412 patients in the primary analysis group, one patient received neoadjuvant therapy only, 82 patients did not have available hormonal data at first surgery and a further 73 patients could not be accurately identified as being in either the luteal or follicular phase of their menstrual cycle. Two hundred and fifty-six patients (62.1%) were therefore included in analysis. Figure 3 displays 3-year OS from first surgery for the two menstrual cycle phases considered here. No differences in survival were seen. Under multivariate analyses, menstrual cycle phase as defined above was not found to be an independent predictor of 3-year OS (HR=0.67, 95% CI [0.28, 1.63]). When phase of menstrual cycle was categorised according to Senie and co-workers, Badwe and co-workers and Hrushesky and co-workers using LMP, results were similar and no effect of timing of surgery in relation to phase of menstrual cycle was found (results not displayed).

Three-year DFS from first surgery for primary analysis group patients was 83.4% (95% CI [79.7, 87.0%]). Of the 66 patients who had a recurrence within 3 years of first surgery, 13 patients had a local recurrence, 51 patients had a systemic recurrence as their first recurrence and two patients had both a local and systemic recurrence on the same day. No statistically significant differences in DFS were found between the seven LMP groups (log-rank=0.85, df=6, *P*=0.97). Three hundred and sixty-two patients were included in multivariate analysis, and since no optimal interval of the menstrual cycle was identified in the analysis of OS from first surgery, menstrual cycle defined using LMP was entered into the Cox's PH model as a continuous variable. Menstrual cycle at the time of first surgery was not found to be a statistically significant independent predictor of 3-year DFS (likelihood ratio=0.01, df=1, *P*=0.92) (Table 4). At the 1% significance level, NPI was found to be the only statistically significant factor independently contributing towards DFS (likelihood ratio=40.84, df=1, *P*<0.0001, HR=2.21, 95% CI [1.74, 2.81]). Results of DFS from FTH were again similar.

## DISCUSSION

In this study, timing of surgery within the menstrual cycle had no significant impact on 3-year survival. Our patients were carefully stratified on the basis of regularity of cycles, and in this analysis, only patients who had regular menstrual cycles and who had not taken the oral contraceptive pill within the previous 6 months were included. Menstrual cycle phase was also assessed both by LMP data as well as hormonal data. In addition, the question of whether it is FTH (mammography, cytology or core biopsy) or surgical intervention that is important can be addressed, as menstrual cycle data were collected for each intervention (including further surgery), making this study unique. However, results for survival from FTH were similar to those from first surgery, which may be expected since median time from FTH to first surgical intervention was only 17 days. In 2002, Hortobagyi (2002) reviewed the subject of menstrual cycle phase influence on timing of surgical treatment for breast cancer and concluded that the only reliable method to confirm the hypothesis suggested by Hrushesky *et al* (1989) was a prospective trial in which participants have careful hormone measurements performed at each intervention to determine their menopausal status and the menstrual cycle phase.

**Figure 4:**
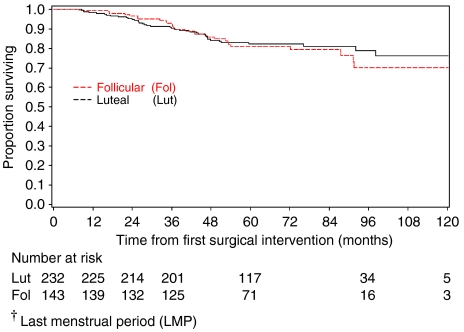
Ten-year OS from first surgery for primary analysis group patients, according to menstrual cycle defined using LMP^†^ and categorised according to Badwe.

Although there were low numbers of deaths in comparison with the NPI expected event rate within 3 years, this study was adequately powered, and a sufficient number of events were observed, to detect an effect as large as that seen by Badwe *et al* (1991). Figure 4 shows OS to 10 years for primary analysis group patients where phase of menstrual cycle is categorised according to the definition of Badwe and co-workers using LMP. This figure shows that the results of no impact of the timing of surgery with respect to the menstrual cycle phase may remain true for longer-term survival; however, it is important to note that not all patients have been followed up for 10 years, and so longer-term follow-up is still required to confirm this.

This study also has the advantage that no change in routine practice was required, or was seen, thus avoiding a potential bias and ethical dilemma of allocating patients to have surgery within one particular phase of the cycle, and hence potentially exposing patients to longer waiting times for surgery in order to coincide with their allocated ‘phase’. In a review of menstrual timing of breast cancer surgery by Hagen and Hrushesky (1998), the design of this study was criticised. The timing of initial analysis was criticised for being after only 2 years of follow-up; however, initial analyses were planned when all patients had at least 3 years of follow-up, and at the time of this analysis the median follow-up of primary analysis patients was 59 months. The study was also criticised for not using prospective menstrual history to complement retrospective menstrual history, for sampling hormone concentrations infrequently, for multiple surgical procedures not being coordinated in time, and for making no attempt to keep clinicians, nurses and patients from biasing menstrual stage assignment. However, this study has in fact collected dates of menstrual bleeding prospectively, which have been used alongside hormone profiles that have been well sampled at each intervention, to define patients’ menstrual cycle phase. Due to UK waiting lists and allocation of surgery timings it was not possible for us to look at rapid succession of surgical interventions, and in this study the median time between FTH and first surgery for primary analysis patients was 17 days. Finally, as discussed above, no change in practice was implemented for this study. All patients have been followed up for 3 years, thus preventing differing follow-up for patients in different menstrual cycle phases, and OS was chosen as the primary endpoint as this will not be affected by clinicians knowing at what stage surgery took place and therefore having differing follow-up intensities between the menstrual cycle phases.

There has been no convincing biological hypothesis put forward by those who have found a difference in survival. Many factors are known to vary within the menstrual cycle, including oestrogen receptor (ER) concentration (Pujol *et al*, 1998). The incidence of vascular invasion has been shown to be higher in tumours removed in the follicular phase (Fentiman, 2002), as have levels of insulin like growth factor, interleukin-2, natural killer (NK) activity and various gene changes (Souza *et al*, 2001). Two groups have examined the prognostic impact of progesterone serum levels based on the hypothesis that resection within the luteal phase gave better outcome. Badwe *et al* (1994) showed no difference for node negative patients, but a significant survival advantage was observed for a small group in whom a high serum progesterone level was reported at the time of resection.

Why were the early retrospective studies so positive? They came from institutions with a good record of prospective collection of data and where patients were dealt with in a very standardised fashion. At Guy's Hospital, the FTH was often an excision biopsy, with definitive surgery carried out at a later stage. Details on adjuvant therapy are scarce, but at the time of these studies many patients did not receive chemotherapy or adjuvant tamoxifen. In our study, 97.6% of patients received adjuvant therapy, with approximately 70% receiving chemotherapy and/or tamoxifen and 80% of patients receiving radiotherapy. Modern chemotherapy suppresses ovarian function, with cessation of menses in many patients. This will depend on the type of regime used and the age of the patient. It could therefore be hypothesised that predictive factors such as menstrual cycle phase identified in these early studies are now overruled by adjuvant therapy use.

At the time the ITS study was started it was not routine practice to measure the ERα status of all breast cancers. It has since been recognised that ERα status and markers of its functionality such as progesterone receptor (PR) provide important prognostic information, are predictive of response to anti-oestrogen therapy and broadly delineate potentially different types of breast cancers (Early Breast Cancer Trialists’ Collaborative Group, 1998). The Surgical Timing And Menstrual Periods study was set up as a sub-study to ITS in order to determine the ERα and PR status of patients registered onto ITS. Longer-term survival subgroup analysis will incorporate these data to assess whether differences in survival according to the timing of interventions with respect to menstrual cycle phase are apparent when accounting for ERα and PR status.

To conclude, timing of breast cancer surgery in relation to the menstrual cycle phase had no significant impact on 3-year survival in this study. This may be due to almost all patients receiving some form of adjuvant therapy. Longer-term analysis will be performed at 10 years when all patients have reached this follow-up. This will allow us to obtain a definitive answer to the controversial question of the effect of timing of surgery with respect to patients’ menstrual cycle phase on prognosis.

## Figures and Tables

**Table 1:** Baseline characteristics of the 412 patients in the primary analysis group

	***n***	**%**
Patients who had mammography	364	88.3
Patients who had fine-needle aspiration	365	88.6
Patients who had a core biopsy	55	13.3
		
Age at first surgery (years) – median (Q1, Q3)	43	(40, 46)
(min., max.)		(22, 54)
		
*LMP* *group at first surgery*		
0–4 days	64	15.5
5–9 days	70	17.0
10–14 days	61	14.8
15–19 days	62	15.0
20–24 days	71	17.2
25–29 days	39	9.5
30+ days	31	7.5
Unidentifiable^a^	14	3.4
		
*Type of first surgery*		
Excision biopsy	74	18.0
Lumpectomy	197	47.8
Mastectomy	138	33.5
Axillary procedure only	1	0.2
Neoadjuvant therapy	1	0.2
Missing	1	0.2
		
*Type of axillary procedure at first surgery*		
None	100	24.3
Sample	69	16.7
Clearance	243	59.0
		
Further surgery required	134	32.5
		
*Adjuvant treatment received*	402	97.6
Chemotherapy given	278	69.2
Hormone therapy given	278	69.2
Radiotherapy given	320	79.6
		
*Histological grade*		
I	59	14.3
II	181	43.9
III	154	37.4
Missing	18	4.4
		
Tumour size (mm) – median (Q1, Q3)	20	(15, 30)
(min., max.)		(0.5, 150)
		
Number of nodes sampled – median (Q1, Q3)	11	(7, 16)
(min., max.)		(0, 36)
		
*Nodal status*		
Positive	208	50.5
Negative	193	46.8
Missing	11	2.7
		
*NPI* *prognostic groups – three categories*		
Good	110	26.7
Moderate	176	42.7
Poor	95	23.1
Missing	31	7.5

LMP=last menstrual period; NPI=Nottingham Prognostic Index.

a Patients whose LMP date or surgery date is missing, or who had neoadjuvant therapy, are classed as having an unidentifiable LMP group.

**Table 2:** Number of deaths and censored values for univariate 3-year OS from first surgery analysis according to the seven LMP groups

**LMP group**	**Number of patients**	**Died**	**Alive^a^**	**Lost to follow-up^b^**	**Overall survival at 3 years**
0–4 days	64	6	56	2	90.3
5–9 days	70	5	62	3	92.6
10–14 days	61	4	53	4	93.3
15–19 days	62	6	53	3	90.1
20–24 days	70	7	60	3	89.7
25–29 days	39	4	33	2	89.5
30+ days	31	4	25	2	86.9
					
Total	397	36	342	19	90.7

LMP=last menstrual period; OS=overall survival.

a Patients censored at 3 years.

b Patients censored before 3 years (at the time they were last known to be alive).

**Table 3:** Results of multivariate analysis of 3-year OS from first surgery, menstrual cycle according to LMP incorporated in its continuous form

	**Estimate**	**Standard error**	**Hazard ratio [95% confidence interval]**	**df**	**Likelihood ratio statistic**	***P*-value**
Nottingham Prognostic Index	0.85	0.16	2.34 [1.73, 3.18]	1	29.77	<0.0001
Hormone therapy (yes *vs* no)	0.65	0.41	1.91 [0.85, 4.30]	1	2.66	0.10
Radiotherapy (yes *vs* no)	−0.22	0.47	0.80 [0.32, 2.02]	1	0.21	0.65
Chemotherapy (yes *vs* no)	−0.64	0.51	0.53 [0.19, 1.43]	1	1.45	0.23
						
Type of first surgery				2	3.12	0.21
Mastectomy *vs* excision biopsy	1.08	0.75	2.93 [0.67, 12.87]			
Lumpectomy *vs* excision biopsy	0.63	0.78	1.87 [0.40, 8.68]			
Day of menstrual cycle	0.02	0.01	1.02 [0.995, 1.042]	1	2.13	0.14

LMP=last menstrual period; OS=overall survival.

**Table 4:** Results of multivariate analysis of 3-year DFS from first surgery, menstrual cycle according to LMP incorporated in its continuous form

	**Estimate**	**Standard error**	**Hazard ratio [95% confidence interval]**	**df**	**Likelihood ratio statistic**	***P*-value**
Nottingham Prognostic Index	0.79	0.12	2.21 [1.74, 2.81]	1	40.84	<0.0001
Hormone therapy (yes *vs* no)	0.17	0.29	1.19 [0.67, 2.10]	1	0.35	0.55
Radiotherapy (yes *vs* no)	0.09	0.38	1.09 [0.52, 2.29]	1	0.06	0.81
Chemotherapy (yes *vs* no)	−0.86	0.37	0.42 [0.21, 0.88]	1	4.87	0.03
						
Type of first surgery				2	0.30	0.86
Mastectomy *vs* excision biopsy	0.01	0.44	1.01 [0.43, 2.38]			
Lumpectomy *vs* excision biopsy	−0.15	0.44	0.86 [0.36, 2.04]			
Day of menstrual cycle	0.001	0.01	1.00 [0.98, 1.02]	1	0.01	0.92

DFS=disease-free survival; LMP=last menstrual period.
